# The Meaning in Life in Suicidal Patients: The Presence and the Search for Constructs. A Systematic Review

**DOI:** 10.3390/medicina55080465

**Published:** 2019-08-11

**Authors:** Alessandra Costanza, Massimo Prelati, Maurizio Pompili

**Affiliations:** 1 Department of Psychiatry, Faculty of Medicine, University of Geneva (UNIGE), 1206 Geneva, Switzerland; 2 Department of Psychiatry, ASO Santi Antonio e Biagio e Cesare Arrigo Hospital, 15121 Alessandria, Italy; 3 Department of Neurosciences, Mental Health and Sensory Organs, Suicide Prevention Center, Sant’Andrea Hospital, Sapienza University of Rome, 00185 Rome, Italy

**Keywords:** suicide, suicidal behavior, suicidal ideation, suicide attempt, meaning in life, suicide protective factors, suicide risk

## Abstract

*Background and Objectives:* Research on suicidal behavior (SB) has frequently focused more on risk factors than protective factors. Since the historic works of Viktor E. Frankl, who inquired how some Nazi concentration camps prisoners maintained their will to live though confronted with pervasive absurdity, Meaning in Life (MiL) has been interpreted as a potent resiliency factor. MiL then declined along a multitude of theoretical perspectives and was associated with various functioning domains of the individual. Surprising, few studies investigated the role of MiL on SB. We aimed to review and synthetize current literature on possible associations between MiL and SB, which included suicidal ideation (SI), suicidal attempts (SA), and completed suicide, focusing on two MiL constructs (the presence of MiL and search for MiL) from the Michael F. Steger’s recent conceptualization. *Material and* *Methods:* A systematic strategy following PRISMA guidelines was used to search for relevant articles in Pubmed/MEDLINE, Scopus, PsycINFO, and ScienceDirect (January 1980–February 2019) and yielded 172 articles, 37 of which met our inclusion criteria. *Results:* MiL emerged as a protective factor against SI, SA, and completed suicides, directly or through mediation/moderation models with other SB-related variables. When distinguishing the presence of MiL and the search for MiL, a consensual protective impact was described for the former. Data for the latter were less consistent but rather oriented towards a non-protective impact *Conclusions:* These findings could have clinical repercussions for SB prevention, in both suicide risk assessment refinement and psychotherapeutic interventions. Further research is needed to examine the dynamic interplay of the two constructs.

## 1. Introduction

Research on suicidal behavior (SB) has frequently focused on suicide risk factors. In contrast, elements that can buffer stressors and protect an individual from SB have received less attention [1]. Historically, exploration into the adaptive and life-maintaining characteristics of non-suicidal people was originated by Viktor E. Frankl, who attempted to elucidate how some Nazi concentration camp prisoners were able to maintain the will to live and which subjective reasons protected them from a pervasive sense of absurdity [2]. He observed that individuals with a “will of meaning” (*Der Wille zum Sinn*) had the best chance of survival [2]. On this basis, Frankl elaborated the discovery path of Meaning in Life (MiL) against the “existential vacuum” as arising from three possible assumptions, uniquely concerning the human condition: (1) creativity (related to the sense of realization of an individual), (2) perception and a search for beauty (in relation to a sense of authenticity towards certain situations or encounters), and (3) the effort of an individual in trying to find a way to determine one’s interior attitude, even when overwhelmed by miserable circumstances or unavoidable suffering [2].

Since Frankl’s initial observations, Meaning in Life (MiL) has been described from a multitude of theoretical perspectives. One primary distinction has been made between a “global or existential” meaning and a “situational or specific” meaning, thereby discerning individuals’ fundamental assumptions from meaning in the context of a particular environmental encounter [3,4,5,6]. In this latter area, the integrated model of “meaning-making”, articulated by Crystal L. Park, is of particular interest [4]. In addition to the distinction between “global” and “situational” meaning, Park proposed the evaluation of “meaning-making efforts” and “meaning made”, inscribing the possible effects of all these subconstructs in a meaning-making process aimed at adjusting one’s experiences of events that are greatly discrepant with one′s larger beliefs, plans, and desires [4].

In the recent psychological literature, Michael F. Steger has proposed that the greatest consensus in the conceptualization of MiL can be centered on two dimensions: “coherence”, or a sense of the comprehensibility and self-concordant ability of making sense in one’s life, and “purpose”, or a sense of core goals, aims, and direction in life [7]. A third facet, “significance”, which focuses on values, worth, and the importance of one’s life, is receiving increasing attention [7]. “Coherence” refers to the cognitive component of MiL, focusing on the perception that stimuli are predictable and conform to recognizable personal patterns that transcend chaos. “Coherence” would be especially activated in situations where meaning is disrupted and the individual experiences distress and the related necessity to construct or reconstruct a framework to understand life [7]. While “purpose” is sometimes used synonymously with MiL, it should be explicitly considered separately from the general sense of MiL and understood as one of its components (the motivational one), based on one’s goals and enthusiasm in life (e.g., spirituality and religiousness were shown to be correlated to MiL but not to “purpose”, while optimism was correlated to “purpose” but not to MiL) [7]. “Significance” constitutes the evaluative component for MiL as it relates to how important, worthwhile, and inherently valuable one’s life as a whole feels beyond trivial or momentary elements [7]. Both “purpose” and “significance” are value-laden concepts, but they differ in two essential aspects, based on their primary motivational versus evaluative nature. “Purpose” is about finding valuable goals future-oriented, while “significance” is about finding value in life, including the past, present, and future [7]. When all three components are taken together, a definition for MiL emerges from “the web of connections, interpretations, aspirations, and evaluations” that “(1) make our experiences comprehensible, (2) direct our efforts toward a desired future, and (3) provide a sense that our lives matter and are worthwhile” [7,8].

Steger’s model divides MiL into two constructs: the presence of MiL and the search for MiL [9]. These two constructs were found not to be mutually exclusive [10]. The presence of MiL is uniformly thought to be beneficial for various functional aspects of life, including adaptive resources, overall psychological well-being, and positive affects [11]. By contrast, the search for MiL appears more controversial. Some researchers consider MiL the essence of human motivation [12,13], while others find it a dysfunctional sign that meaning has been frustrated or lost [14,15]. In a third perspective, the search for MiL can have either healthy or non-healthy connotations depending on the motivational [3] and personal characteristics of the individual [16]. Addressing this issue from a typological perspective, MiL profiles resulting from a combination of high scores in the presence of MiL and low scores in the search for MiL have been associated with better adjustment outcomes [17,18]. Both constructs are highly stable over time, suggesting that MiL more accurately reflects a trait aspect than a state aspect of individual functioning [18]. 

From a clinical viewpoint, exploring MiL in suicidal patients during psychiatric interviews would personalize and improve their SB risk assessments [19,20]. Interventions targeting MiL have also been found effective in reducing suicide risk [21] and represent a promising therapeutic opportunity [19,20,22].

Few studies have explored MiL in individuals presenting SB. With this review, we aimed to examine existing published data to investigate possible associations between MiL and suicidal ideation (SI), suicide attempts (SA), and completed suicide. Particular attention was given to studies that distinguished between the roles of the presence of MiL and the search for MiL. We hypothesized that MiL has a protective effect on SI, SA, and completed suicide. Specifically, for works that addressed the two constructs of MiL, we hypothesized that the presence of MiL would have a protective effect. The exiguity and contradictory nature of the data on the search for MiL did not allow us to formulate a specific hypothesis but only to perform exploratory analyses.

## 2. Methods

This review was conducted according to the Preferred Reporting Items for Systematic Reviews and Meta-Analyses (PRISMA) guidelines [23] and the Cochrane collaboration guidelines [24]. 

### 2.1. Information Sources and Search Strategy

We performed a systematic search in four major electronic databases comprising medical and social science research (PubMed/MEDLINE, Scopus, Science Direct, and PsychINFO) for relevant titles and abstracts published between January 1980 and February 2019. Additional articles were retrieved from the reference lists of relevant articles and from published reviews. The following combined search queries of free-text terms and exploded Medical Subject Headings (MeSH) terms were used for the Pubmed/MEDLINE database: “Meaning in Life” AND “Suicidal ideation” [MeSH] OR “Suicide, attempted” [MeSH] OR “Suicide” [MeSH] OR “Suicidal Behavior” OR “suicidality.” This search strategy was adapted for use with the other databases.

### 2.2. Eligibility Criteria

Articles that explicitly mentioned a potential association between MiL and SB (OR suicidal ideation OR suicide attempts OR completed suicides) in non-clinical and clinical samples were included. When a title or abstract seemed to describe an eligible study, the full-text article was obtained and carefully examined to assess the study’s relevance for our review. Our exclusion criteria were: (1) articles published before 1980, (2) articles with abstracts that did not directly mention an investigation into a potential association between MiL and SB, (3) articles not published in peer-reviewed journals, (4) articles not published in English, and (5) meta-analytic, systematic, or narrative reviews, or book chapters.

### 2.3. Study Selection and Data Collection

Studies were independently reviewed by two authors (A.C. and M.Pre.) using a two-step process. First, screening and selection were performed based on the article’s title and abstract. Second, further screenings and selections were performed on retrieved full-text articles. A data extraction spreadsheet was developed [23]. The data were extracted by one author (A.C.) and supervised by another (M.Pre.). The data elements of interest were author(s), publication year, study design, sample characteristics (population type, sample size, and psychiatric diagnosis when appropriate), instrument(s) used to assess MiL, and the impact on SB-related variables (SI, SA, completed suicide, other SB-related variables, and/or main commentaries). At any stage of the article selection and data collection processes, disagreements were resolved through discussion with the senior reviewer (M.Pom.), who also independently read all the articles. 

### 2.4. Summary Measures

As with previous studies [25,26], we assessed the selected studies for quality using the following criteria: (1) the representativeness of the sample for the general population, (2) the presence and representativeness of a control group, (3) the presence of longitudinal follow-up, (4) the evidence-based measures of MiL (e.g., a Meaning in Life Questionnaire, Purpose in Life Questionnaire, or other psychometric instruments), (5) the presence of raters who independently identified MiL, (6) the statistical evaluation of inter-rater reliability, and (7) evidence-based measures of SI or SA (e.g., a Suicidal Ideation Questionnaire, Suicide Risk Scale, Beck Hopelessness Scale, or other psychometric evaluation). A score of 0–2 points was attributed to each item, yielding a quality score ranging from 0 to 14. Studies were divided into 3 groups: (1) good quality (10–14 points), if most or all the criteria were fulfilled, or, where they were not met, the study conclusions were deemed very robust; (2) moderate quality (5–9 points), if some criteria were fulfilled or the study conclusions were deemed robust; or (3) low quality (0–4 points), if few criteria were fulfilled or the study conclusions were not deemed robust. Caution was adopted in interpreting the findings from the low-quality studies. Disagreements between reviewers were resolved by consensus.

## 3. Results

### 3.1. Included Studies

**Figure 1:**
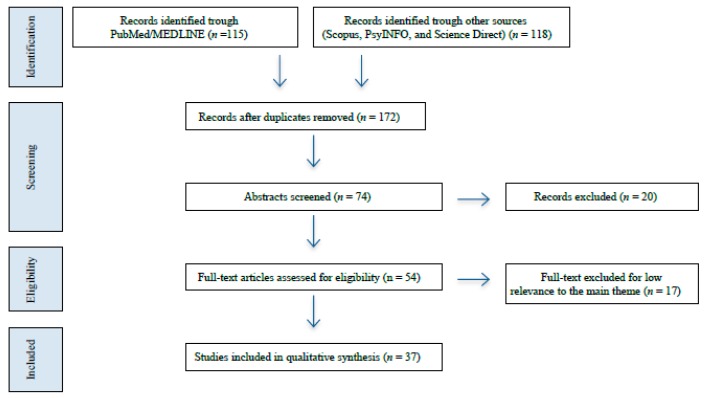
Flowchart for the search and selection process [23].

After removing 61 duplicates, a total of 172 potentially relevant articles were found. Figure 1 shows the flow through the identification, screening, and assessment of eligibility. Studies were excluded either because the exclusion criteria were met or because of low relevance compared to our primary theme. Finally, 37 studies met our inclusion criteria and were included in our qualitative synthesis for this review.

### 3.2. Characteristics of Included Studies (Study Designs and Samples)

The characteristics of the included studies are shown in Table 1 and Table 2. In total, 24 studies were cross-sectional, nine were prospective and longitudinal, and five were qualitative (one was classified as both cross-sectional and longitudinal). Additionally, 20 were conducted using non-clinical populations (Table 1 and Table 2), and 17 were conducted using psychiatric and non-psychiatric clinical populations (Table 1 and Table 2). Among the non-clinical populations, undergraduate students and adolescents were the most frequently represented groups (*n* = 12) followed by elderly people (*n* = 7). Eight studies included individuals aged 12–70 years, one included only adults, and one included individuals aged at least 20 years and was based on the general population participating in the Nord-Trøndelag Health Study in Norway (the HUNT I cohort during 1984–1986 and the HUNT II cohort during 1995–1997). The specific studied populations were military personnel and veterans (*n* = 3), Chinese professional employees (*n* = 1), homeless people (*n* = 1), and disadvantaged African American female survivors of a recent SA (*n* = 1). Among studies performed using clinical populations, mood disorders were the most specifically addressed psychiatric diagnoses (*n* = 6), followed by borderline personality disorders (*n* = 3). Finally, five studies were performed based on various mental disorders (including mood and anxiety disorders, borderline and avoidant personality disorder, and post-traumatic stress disorder (PTSD), eating disorders, substance use disorder, and psychotic spectrum disorder), two were based on PTSD alone, and one was based on eating disorders. The only clinical non-psychiatric population studied was the population diagnosed as HIV-positive.

Of the studies that addressed the impact of the two MiL-distinct constructs (the presence of MiL and the search for MiL) (*n* = 5; Table 2), one was both cross-sectional and prospective, two were cross-sectional, and two were longitudinal and prospective. No qualitative studies were represented in this group. Three studies were conducted among non-clinical populations (two among undergraduate students and one among soldiers returning from deployment; Table 2), and two were conducted among clinical populations (one among patientss diagnosed with severe PTSD/depression and one among those diagnosed as HIV-positive; Table 2).

Interest in this research subject has increased over very recent years (32 of the included studies were published in 2013 or later). This was particularly evident among studies conducted using clinical populations (12 of these 17 studies were published in 2015 or later). Most of these (*n* = 7) were performed using Spanish clinical samples by Marco and colleagues. Of those performed using non-clinical samples of adolescents/undergraduate students, nearly half (5 of 12) were performed using Israeli samples, and four of these were performed by Wilchek-Aviad and colleagues. Notably, all the studies that focused on the impact of the two MiL-distinct constructs (presence of MiL and search for MiL) were published in 2013 or later, and all but one were published in 2016 or later. Notably, two of the three studies using samples of military personnel and veterans focused on these two constructs.

### 3.3. Quality Assessments

Based on our quality scoring system, the mean score of the included studies was 7.2. Most (*n* = 16) were of moderate quality, followed by those of low quality (*n* = 14) and those of good quality (*n* = 7).

### 3.4. Primary Findings

#### 3.4.1. Studies of Associations between MiL and SB-Related Variables

The primary findings of the included studies that aimed to elucidate associations between MiL and SB-related variables are shown in Table 1. In these studies, MiL was assessed using either the Purpose in Life test [60] or its 10-item shortened version [61], the Geriatric Suicide Ideation Scale, the Perceived MiL subscale [62], the Experienced Meaning in Life instrument [63], the Life Regard Index [64,65], the Sense of Coherence Scale [66], the Crisis of Meaning Scale [67], the Meaningful Life Measure [68], the Spiritual Well-Being Scale (including the Existential Well-Being subscale and the Religious Well-Being subscale [69]), and qualitative analysis. Steger’s Meaning in Life Questionnaire (MLQ) and its three-item shortened version [70] were also utilized, but without separate measures for the presence of, and search for, constructs.

Most studies investigated the impact of MiL on SI, and an inverse association was reported as direct [22,27,29,34,36,39,41,42,43,45,48,50,53] and/or through mediation and moderation models. MiL was found to mediate the relationships between SI and a variety of factors: stress/coping (via an inverse effect on depression) [1], “Reason for Living” [35], psychological strain [40], and satisfaction in life [43]. It was also found to mediate the relationship of both SI and hopelessness with PTSD severity [54]. Additionally, MiL was found to moderate associations between SI and depression [43] and between risk factors for suicide and hopelessness [19,49]. Both the mediation and moderation effects of MiL was found for gratitude and grit, which were shown to work synergistically to enhance MiL and confer resiliency to suicide by increasing MiL [31]. In bullying, a mediation model was reported in the female population, in which MiL explained how victimization can lead to SI, while a moderation model was reported in the male population, in which MiL attenuated the victimization effect on SI [32]. 

An inverse association between MiL and SA has been found in many studies [20,27,39,52]. Similar to the findings with SI, MiL was found to mediate the relationship between SA and stress/coping (via an inverse effect on depression) [1]. However, a moderation model was not described.

Only 1 study showed an inverse association between MiL and completed suicides. This result was based on two large cohorts of the general population [30]. The association remained significant after controlling for a common mental disorder that emerged during the survey. Unfortunately, only a published abstract was available for this study.

Inverse associations were also reported between MiL and suicidal tendency or suicidal potential (a measure accounting for depression, anxiety, and emotional state) [33,36,38] and between MiL and suicide risk (taking into account general suicide risk factors) [46].

Mental pain was inversely related to MiL [28] (study 2). Two variables of the Interpersonal-Psychology Theory of Suicide (IPTS) [71,72] were explored in relation to MiL. “Perceived burdensomeness” could contribute to suicide morbidity and mortality by eroding MiL [44], and “thwarted belongingness” was associated with more lethal methods for SA and increased SA [47]. Although not formally related to the IPTS, two additional context-relational variables were tied to meaninglessness in the SI experience: aloneness [45] and missing (or perceived missing) connectedness [42]. MiL, but neither religiosity [41] nor religious well-being [53,54], acted as protective factors against SI [41,53] and mediated the relationship between PTSD severity and both hopelessness and SI [54].

#### 3.4.2. Studies of Associations between the Presence of MiL and the Search for MiL and SB-Related Variables

The primary findings of the included studies that aimed to elucidate associations between the presence of MiL and the search for MiL and SB-related variables are shown in Table 2. These two constructs of MiL were assessed using Steger’s MLQ, which consists of a self-reported 10-item inventory measuring the extent to which individuals feel their lives are meaningful (the presence of MiL, five items) and the extent to which they are actively seeking meaning (the search for MiL, five items) [70]. 

##### Presence of MiL

The presence of MiL predicted decreased SI over time and lowered the lifetime odds of SA among undergraduate students in the longitudinal prospective analyses (with an average follow-up of 2 months) and cross-sectional analyses, respectively, from the same study [55]. These findings remained significant above and beyond the effects of low levels of psychopathology (depression and anxiety symptoms) and high levels of protective factors (gratitude and social support) [55]. In a study examining the factors related to changes in suicide risk among soldiers returning from deployment in Iraq and Afghanistan, surveyed 6 and 12 months following their return, the negative association between the presence of MiL and suicide risk became greater, but this finding was not statistically significant [56]. In this study, suicide risk was assessed using the Suicide Behavior Questionnaire-Revised, which includes four dimensions of SI and SB (lifetime SI and/or SA, frequency of SI over the past 12 months, the threat of SA, and self-reported likelihood of SB in the future) [73]. Among military personnel and veterans with severe PTSD and depression, the presence of MiL negatively mediated the relationship between PTSD or depression and the trajectory leading from the emergence of SI to SA [58]. The presence of MiL was found to moderate the association between depressive symptoms and SI but not the association between depressive symptoms and SA in HIV-positive patients 6 to 12 months following diagnosis [59]. Data for completed suicides were not available.

The presence of MiL mediated the relationships between MiL and the IPTS variables of “perceived burdensomeness” and “thwarted belongingness” and SI [55]. Additionally, the experimentally enhanced presence of MiL conferred resilience to perceived “burdensomeness and “thwarted belongingness” [57]. Both studies were performed using undergraduate students [55,57].

##### Search for MiL

The search for MiL predicted a decreased SI over time, but with a lower threshold for statistical significance than the presence of MiL, and it did not predict the lifetime odds of SA among undergraduate students [55]. In particular, the search for MiL was shown to predict post-deployment suicide risk, which increased among soldiers returning from deployment in Iraq and Afghanistan based on surveys administered 6 and 12 months following their return [56]. The strength of the association between initial depressive symptoms and becoming a high post-deployment suicide risk was diminished after accounting for the search for MiL. The search for MiL did not negatively mediate the relationship between PTSD or depression and the trajectory from SI to SA among military personnel and veterans [58]. Data for completed suicides were not available.

The search for MiL did not mediate the relationships between MiL and “perceived burdensomeness” or “thwarted belongingness” and SI [55], and it was not investigated in experimentally-enhanced conditions of interpersonal adversity [57] among undergraduate students [55,57]. 

## 4. Discussions

With this review, we have aimed to investigate the associations between MiL and SB, including SI, SA, and completed suicide. These associations have been examined in studies that considered MiL and those that distinguished the two MiL constructs (the presence of MiL and the search for MiL), according to Steger’s model. We placed a particular focus on the latter.

The included studies consensually showed the protective impact of MiL on SI, SA, and completed suicide, whether conducted using clinical or non-clinical populations. In studies distinguishing the two MiL constructs (the presence of MiL and the search for MiL), a unanimous protective impact on SB-related variables was described only for the former. Correlations between the latter and SB-related variables were less consistent but were globally oriented towards a non-protective impact.

However, comparisons within the first group of studies are limited by an important heterogeneity in the neuropsychological assessment of MiL (Purpose in Life test, Geriatric Suicide Ideation Scale, Perceived MiL subscale, Experienced Meaning in Life instrument, Life Regard Index, Sense of Coherence Scale, Crisis of Meaning Scale, Meaningful Life Measure, Spiritual Well-Being Scale, and MLQ), presupposing different MiL conceptualizations that can overlap but cannot be considered synonymous [3,5,6]. By contrast, only one instrument, the MLQ [70], was utilized in the second group of studies, implying a more uniform MiL conceptualization upstream that includes the distinction between the presence of MiL and search for MiL.

Non-unanimous findings of the role of the search for MiL in SB reflect the debate surrounding the role of search for MiL in other functional aspects of individuals. In their longitudinal analysis of undergraduate students, Kleiman and Beaver [55] found that both the presence of MiL and the search for MiL predicted decreased SI over time, even if the statistical effect of the search for MiL was more marginal. Their cross-sectional analysis at baseline revealed, instead, that only the presence of MiL was associated with lower lifetime odds of SA. They argued that the search for MiL can only act as a protective factor against less severe manifestations of suicidality, which they supposed to be SI compared to SA. Their findings were especially interesting because the presence of MiL was shown to confer suicide resiliency independent of low levels of risk factors, such as psychopathology (depression and anxiety symptoms), and protective factors (gratitude and social support). However, they recognized a number of study limitations, which included the use of a non-clinical population, a large majority of female representatives in their sample, self-reporting of anxiety and depression symptoms, and a relatively small number of participants with a SA history, leading to an underpowered analysis based on SA [55].

The two studies performed using military personnel and veterans concluded only partially concordant findings on the beneficial versus deleterious impacts of the presence of MiL and search for MiL. Kim and colleagues [56] surveyed the suicide risk among soldiers returning from Iraq and Afghanistan twice during their first year post-deployment, observing that suicide risk increased significantly between the 6th and 12th months. Greater levels of search for MiL and perceived stress were associated with becoming a high suicide risk. The strength of the association between initial depressive symptoms and suicide risk was attenuated after accounting for these measures. On the other hand, they found a consistent negative association between the presence of MiL and becoming a high suicide risk post-deployment, but this relationship was not statistically significant [56]. The primary finding made by Sinclair and colleagues [58] in a study of military personnel and veterans with elevated levels of PTSD and depression was that the presence of MiL negatively mediated the relationship between PTSD or depression and the trajectory from SI to SA, particularly during acute experiences of mood disturbances. They estimated that this mediation was crucial for explaining why some military personnel and veterans do not become suicidal despite the correlation between their clinical conditions (PTSD/depression) and suicide risk. Contrary to their expectations, they found that search for MiL did not show the same mediation effect, suggesting that the active pursuit of MiL is not central to the trajectory from SI to SA [58].

In a clinical non-psychiatric cohort of HIV-positive patients surveyed 6 and 12 months after diagnosis (a population at high risk for both SI and SA), Lu and colleagues [59] reported that depressive symptoms were the primary predictors of changes in both SI and SA. Among the examined psychosocial factors, they found that only the presence of MiL buffered the relationship between depressive symptoms and SI. Neither the presence of MiL nor search for MiL showed a similar moderating effect on the relationship between depressive symptoms and SA [59].

Finally, Kleiman and Beaver [55] and Collins and colleagues [57] described the protective effect of the presence of MiL (but not the search for MiL) deployed on SI through two IPTS variables (“perceived burdensomeness” and “thwarted belongingness”) described by Joiner and colleagues [71,72], in two cohorts of undergraduate students. According to IPTS, “perceived burdensomeness” and “thwarted belongingness” combine to produce SI [71,72]. The findings in the student samples mentioned above [55,57] were valorized because they potentially offered an additional way to explain and weaken the SI risk conferred by interpersonal adversities. However, both constructs were assessed in the former study [55], but only the presence of MiL was investigated (and manipulated in experimental conditions) in the latter study [57]. Inspired by IPTS, which marked a crucial turning point in suicide theories by distinguishing the emergence of SI from the progression from SI to SA (mediated in this case by the coexistence of a third variable, the “acquired suicidal capability”), another “Ideation-to Action” framework was proposed by Klonsky and May: the Three-Step Theory of Suicide (3ST) [74]. This framework posits that SI first results from the combination of pain (usually psychological pain) and hopelessness. Second, among those experiencing both these conditions, connectedness is a key protective factor against SI escalation. Third, the progression from SI to SA is facilitated by dispositional, acquired, and practical contributors to the capacity to make SA [74]. In the theories of Joiners and colleagues and Klonsky and May, connectedness represents an essential resource and a fundamental step in the passage from suicidal ideation to suicidal action. As previously noted [20], connectedness can be considered similar to MiL in its reference to one′s attachment to a work, to a role, or to shared projects and interests that keep one invested in living. Thus, their negative predictions for the role of SI could also be conceptually linked.

Taken together, these findings show that exploring the presence of MiL and the search for MiL contribute to refining the SB risk assessment. By widening the boundaries of diagnostic interviews to these two constructs, new entry points to SI could possibly be determined in both clinical and non-clinical populations [55], but particularly in populations that (1) can have silent/occult SI and (2) can abruptly experience out-of-the-ordinary experiences (e.g., veterans) [22]. 

Moreover, the presence of MiL and the search for MiL could constitute a useful target for psychotherapeutic interventions designed to decrease SB risk. These two constructs can be dealt with from different perspectives. Therapeutic modalities were initially systematized according to the classical assumptions of the existential–humanistic Logotherapy of Viktor E. Frankl [75]. Subsequently, Meaning-Centered Counseling, consisting of a cognitive-behavioral reformulation of Logotherapy [76], and Meaning-Centered Counseling and Therapy, consisting of an eclectic model that integrates different theories with MiL as its central and unifying theme [77], were proposed. Finally, a program aimed at achieving meaningful personal goals has been described [21], which, in patients with a borderline personality disorder, could be articulated with Dialectical Behavioral Therapy [20,49]. Although not specifically, the first three models [75,76,77] address both constructs, while the last model [20,21,49] is more focused on the presence of the Mil construct. Considering all these previous, precious, contributions (and after we have acquired more knowledge on the dynamic interplay of the two constructs), a specific approach for both the presence of Mil and the search for MiL could provide a promising psychotherapeutic area of study.

## 5. Limitations

This review has several limitations. First, it is based on studies of various natures. In qualitative and retrospective studies, clinical variables are not always readily available. Second, the studies investigated very heterogeneous populations. Some were conducted using non-clinical individuals of various ages and socio-professional conditions, while others used clinical populations with various psychiatric diagnoses, who were either outpatients or inpatients. Some studies also considered mixed samples of patients belonging to different age groups with various psychiatric diagnoses and in various stages of a given disorder. Third, these studies used varied instruments to examine the possible links between SB and the constructs in question. Furthermore, the SB risk was sometimes evaluated using differing methods. Given these factors, the data were often difficult to make generalizable.

We did not carry out a meta-analysis because the data from most of these studies did not permit such an approach. The studies used different measures and had differing outcomes, assessed patients at differing time points, and had several methodological problems, which often made the results difficult to interpret. Moreover, most of the studies considered nonhomogeneous samples, analyzed only a few variables, and did not include a control group. Finally, there are a limited number of articles in the literature concerning the topic of this review, particularly when distinguishing between the presence of MiL and the search for MiL.

## 6. Conclusions

In conclusion, considering the important limitations stated, MiL nonetheless emerges as a protective factor against suicidality. This has been demonstrated particularly for the MiL construct presence of MiL on SI and its inclusion among non-clinical psychiatric populations. These findings have clinical repercussions on SB prevention in both SB assessment and psychotherapeutic interventions. However, further research is needed to confirm the role of the presence of MiL and to clarify its interplay with the search for MiL, particularly in (1) clinical psychiatric populations (to possibly quantify their impacts on SB risk despite clinical conditions), (2) longitudinally designed cohorts, and (3) studies addressing SAs.

## Figures and Tables

**Table:** (**A**)

Non-Clinical Populations (*n* = 17)								
Author(s)	Study Design	Sample		Instrument Assessing MiL	SB-Related Variables			
		Population	Size (*N*)		SI	SA	Completed Suicide	Other SB-Related Variables and/or Main Commentaries
Edwards and Holden, 2001 [27]	Cross-sectional	Undergraduate students	298	PIL, Sense of Coherence Scale	↓	↓	−	↓ Self-reported likelihood of future SB
Orbach et al., 2003 (study 2) [28]	Cross-sectional	Undergraduate students	98	LRI	−	−	−	MiL inversely related to mental pain
Wang et al., 2007 [1]	Cross-sectional	Undergraduate students	416	PIL	↓	↓	−	Mediation model: MiL mediated relationships between stress, coping, SI, and SA indirectly via an inverse effect on depression
Heisel and Flett, 2008 [29]	Cross-sectional	Elderly	107	GSIS Perceived MiL subscale	↓	−	−	−
Bjerkeset et al., 2010 [30]	Longitudinal prospective	Individuals aged 20+ yr, based on the Norwegian HUNT general population cohort	141,117	Self-reported measure of sense of MiL (n.sp.)	n.sp.	n.sp.	↓	A lower sense of MiL associated with increased suicide risk after controlling for common mental disorders that emerged during the survey
Kleiman et al., 2013 [31]	Longitudinal prospective	Undergraduate students	209	MLQ	↓	−	−	Mediated moderation model: gratitude and grit work synergistically to enhance MiL and confer resiliency to suicide by increasing MiL
Henry et al., 2014 [32]	Cross-sectional	Undergraduate students	2936	3-item MLQ	↓	−	−	Mediation model (female population): MiL could explain how bullying victimization leads to SI; moderation model (male population): effect of victimization on SI was attenuated as MiL increased
Wilchek-Aviad, 2015 [33]	Cross-sectional	Adolescents (Ethiopian immigrant and native-born Israeli)	277	PIL	−	−	−	↓ Suicidal tendencies (measured while accounting for depression and anxiety/emotional state) beyond one’s immigrant and native-born status
Denneson et al., 2015 [22]	Qualitative	Veterans	34	Semi-structured interviews	↓	−	−	
Heisel and Flett, 2016 [34]	Longitudinal prospective	Elderly	126	EMIL, PIL	↓	−	−	−
Heisel et al., 2016 [35]	Longitudinal prospective	Elderly	109	EMIL	↓	−	−	Mediation model: MiL mediated associations between “Reasons for Living” and SI; it also explained the significant unique variance in SI
Wilchek-Aviad and Malka, 2016 [36]	Cross-sectional	Adolescents (Jewish religious and secular)	450	PIL	−	−	−	↓ Suicidal tendency (see above) beyond religiosity
Wilchek-Aviad et al., 2017 [37]	Cross-sectional	Adolescents (having different types of leisure time activities)	450	PIL	↓	−	−	MiL was greatest among adolescents involved in social endeavors, lower among those involved in solitary activities, and lowest among those not involved in any leisure activity
Wilchek-Aviad and Ne’eman-Haviv, 2018 [38]	Cross-sectional	Adolescent girls (disadvantaged at different stages of rehabilitation and normative)	209	PIL	−	−	−	↓ Suicidal potential (equivalent to the suicidal tendency, see above) among normative and disadvantaged adolescent girls residing in boarding schools
Schnell et al., 2018 [39]	Cross-sectional	Undergraduate students	300	Crisis of Meaning Scale	↓	↓	−	Crisis of meaning was distinguished from depression and predicted suicidality in youth independent of depression
Liu et al., 2018 [40]	Cross-sectional	Chinese professional employees	687	MLM	↓	−	−	Mediation model: MiL mediated relationships between psychological strain and SI
Testoni et al., 2018 [41]	Qualitative	Homeless people	55	Thematic and interpretative phenomenological analysis	↓	−	−	MiL was the most important reason for living; when it was considered unworkable, addiction/alcoholism represented a strategy to endure life in the street. Neither religiosity nor meaning of death were protective factors for addiction/alcoholism or SI

**Table:** (**B**)

Clinical Populations (*n* = 15)									
Author(s)	Study Design	Sample			Instrument Assessing MiL	SB-Related Variables			
		Population	Size (*N*)	Psychiatric Diagnosis		SI	SA	Completed Suicide	Other SB-Related Variables and Main Commentaries
Moore, 1997 [42]	Qualitative	Elderly	11	Depression	Hermeneutic analysis	↓	−	−	MiL descriptions were always tied to relational contexts: meaninglessness relative to missing (or perceived to be missing) connectedness
Heisel and Flett, 2004 [43]	Cross-sectional	Adults	49	Various	PIL	↓	−	−	MiL accounted for significant variance in SI—also, a mediation model between satisfaction in life and SI and a moderation model between depression and SI
van Orden et al., 2012 [44]	Longitudinal prospective	Elderly	65	Depression, anxiety	GSIS Perceived MiL subscale	−	−	−	“Perceived burdensomeness” might contribute to suicide morbidity and mortality by eroding MiL
Holm et al., 2014 [45]	Qualitative	Elderly	9	Mood disorder	Hermeneutic analysis	↓	−	−	MiL in the experience of SI was associated with existential aloneness: “Being alone without MiL”
García-Alandete et al., 2014 [46]	Cross-sectional	16–60 yr old	80	Borderline personality disorder	PIL-10	−	−	−	↓ Suicide risk (measured accounting for general suicide risk factors), ↓ depression,↓ hopelessness
van Orden et al., 2015 [47]	Qualitative	Elderly	101	Various	Semi-structured interviews	−		−	“Thwarted belongingness” was associated with more lethal methods and increased re-attempts
Braden et al., 2015 [48]	Cross-sectional	Veterans	110	Depressive disorder	LRI Framework subscale	↓	−	−	The relationship between MiL and SI remained significant after accounting for depressive symptoms, past SA, prior inpatient psychiatric hospitalization, and poor physical health
Marco et al., 2016 [19]	Cross-sectional	13–68 yr old	224	Various	PIL-10	−	−	−	Moderation model: MiL buffered associations between suicide risk factors and hopelessness
Marco et al., 2017a (study 2) [20]	Cross-sectional	13–70 yr old	80	Borderline personality disorder	PIL-10	−	↓	−	MiL was also negatively correlated with other behavioral symptoms of borderline personality disorders, including suicidal threats, high-risk behaviors, drug overdoses, and aggressive behavior
Marco et al., 2017b [49]	Cross-sectional	13–56 yr old	124	Borderline personality disorder	PIL-10	−		−	Moderation model: MiL buffered associations between suicide risk factors and hopelessness
Marco et al., 2017c [50]	Cross-sectional case-control	12–60 yr old	474	Eating disorder	PIL	↓	−	−	Patients with eating disorders had lower MiLs and greater SI than the controls; MiL predicts greater levels of both eating disorder psychopathologies and SI
Pérez Rodriguez et al., 2017a [51]	Cross-sectional	18–60 yr old	150	Various	PIL-10	−	NS	−	Hopelessness (specifically its affective component) differentiated between patients with non-suicidal self-injuries and those with SA but not MiL, which underlies the continuum of self-harm
Pérez Rodriguez et al., 2017b [52]	Cross-sectional	12–60 yr old	348	Various (mainly eating disorder)	PIL-10	−	↓	−	Lower levels of MiL and higher levels of hopelessness, borderline symptoms, and non-suicidal self-injuries were associated with SA in the previous year
Lamis et al., 2018 [53]	Cross-sectional	19–65 yr old	112	Bipolar disorder	SWBS (EWB + RWB)	↓	−	−	Existential MiL but not religious well-being acted as a protective factor against SI among bipolar disorder patients and those who experienced childhood sexual abuse
Florez et al., 2018 [54]	Longitudinal prospective	Disadvantaged African American female survivors of a recent SA	113	PTSD	SWBS (EWB + RWB)	↓	−	−	Mediation model: existential MiL, but not religious well-being, mediated the relationship between PTSD severity and both hopelessness and SI level

Note: MiL = Meaning in Life; SB = suicidal behavior; SI = suicidal ideation; SA = suicide attempt; PIL = Purpose in Life test; LRI = Life Regard Index; GSIS = Geriatric Suicide Ideation Scale; n.sp. = not specified; MLQ = Meaning in Life Questionnaire; 3-item MLQ = 3-item shortened version of the MLQ; EMIL = Experienced Meaning in Life instrument; PIL-10 = 10-item shortened version of the PIL; MLM = Meaningful Life Measure; yr = years; NS = not significant; SWBS = Spiritual Well-Being Scale; EWB = Existential Well-Being subscale; RWB = Religious Well-Being subscale.

**Table:** (**A**)

Non-Clinical Populations (*n* = 3)								
Author(s)	Study Design	Sample		Instrument Assessing MiL	SB-Related Variable			
		Population	Size (*N*)		SI	SA	Completed Suicide	Other SB-Related Variables and/or Main Commentaries
Kleiman and Beaver, 2013 [55]	Cross-sectional and longitudinal prospective	Undergraduate students	670 (cross-sectional analysis); 585 (prospective analysis)	MLQ	↓ SI over time for both presence of MiL and search for MiL (greater effect for presence of MiL; minor effect for search for MiL)	↓ lifetime SA odds for presence of MiL	−	Additional findings: The presence of MiL, but not the search for MiL, mediated the relationship between MiL and the burdensomeness or thwarted belongingness and SI
Kim et al., 2017 [56]	Longitudinal prospective	Soldiers returning from deployment	970	MLQ	↓ for presence of MiL(miao)and ↑ for search for MiL	↓ for presence of MiL and(miao)↑ for search for MiL	−	Suicide risk (including four dimensions of SI and SB): ↑ for the search for MiL; (miao)↓ for the presence of MiL (the latter was described by the authors as consistent but not significant)
Collins et al., 2018 [57]	Cross-sectional	Undergraduate students	93	MLQ (the presence of MiL subscal only)	−	−	−	An experimentally-enhanced presence conferred resilience to the interpersonal adversity (“perceived burdensomeness” or “thwarted belongingness”) implicated in suicide risk

**Table:** (**B**)

Clinical Populations (*n* = 2)									
Author(s)	Study Design	Sample			Instrument Assessing MiL	SB-Related Variables			
		Population	Size (*N*)	Psychiatric Diagnosis		SI	SA	Completed Suicide	Other SB-Related Variables and/or Main Commentaries
Sinclair et al., 2016 [58]	Cross-sectional	Military personnel and veterans	393	Elevated PTSD and depression	MLQ	−	−	−	Mediation model: The presence of MiL, but not search for MiL negatively mediated the relationship between PTSD or depression and the trajectory from SI to SA
Lu et al., 2018 [59]	Longitudinal prospective	HIV-positive patients	113	−	MLQ	↓ for presence of MiL, NS search for MiL	NS for presence of MiL; NS for search for MiL	−	Moderation model: The presence of MiL buffered the relationship between depressive symptoms and SI (no moderating effect between depressive symptoms and SA)

Note: MiL = Meaning in life; SB = suicidal behavior; SI = suicidal ideation; SA = suicide attempt; MLQ = Meaning in Life Questionnaire; PTSD = post-traumatic stress disorder; NS = not significant.
